# Uncommon Complication of Uterine Artery Embolization: Expulsion of Infarcted Myoma and Uterine Sepsis

**DOI:** 10.1155/2016/8695318

**Published:** 2016-03-17

**Authors:** Juliana G. Martins, Dawn Gaudenti, Frank Crespo, Dervi Ganesh, Usha Verma

**Affiliations:** OBGYN Department, Jackson Memorial Hospital, University of Miami, Miami, FL, USA

## Abstract

Uterine leiomyomas are the most common benign tumors in young females and leading cause of hysterectomy. Uterine artery embolization is a safe option for women who wish to retain their uterus. Several complications have been reported including expulsion and sepsis. MRI is a useful pretreatment tool to predict results and outcomes. We report a case of a 44-year-old female with a history of uterine fibroids with the largest one being intracavitary. Patient underwent uterine artery embolization that was complicated by endomyometritis that failed antibiotics, leading to sepsis and hysterectomy.

## 1. Introduction

Uterine leiomyomas are the most common benign pelvic tumors in women over 35 years and they are the leading indication of hysterectomy in the United States, with more than 200,000 procedures performed annually [1–4]. Most of women are asymptomatic; however, 20% may present with symptoms that are either abnormal uterine bleeding or bulk-related symptoms [1, 2].

Hysterectomy has been the traditional treatment for symptomatic fibroids; however it is associated with 1–3% incidence of major complications. Uterine artery embolization (UAE) is a treatment option for uterine fibroids to improve abnormal bleeding and pain/pressure symptoms, indicated for premenopausal woman who failed hormonal management and want to avoid surgery [5–8]. The American College of Obstetricians has recommended UAE as an option for women who wish to retain their uterus [7].

Several complications after UAE have been described in the literature; most of them are not life-threatening; however major complications have been reported, including fatal cases [9–12]. Endometritis and sepsis are rare complications of UAE, with an infection rate of 2%. Early recognition of infection and prompt management are crucial [13–15].

Primary treatment of endometritis includes intravenous fluids and antibiotics. In addition, the necrotic prolapsed fibroid should be removed and the uterine cavity should be evacuated of any necrotic residual tissue. When treatment fails, hysterectomy should be considered without any delay to avoid fatal complication of septicemia and multiorgan failure due to uterine necrosis and sepsis [13–15].

Magnetic resonance imaging (MRI) is an accurate and noninvasive preprocedural modality in women who will undergo UAE since it will allow an appropriate selection of the patients and improve the effectiveness of this modality [5].

According to available literature, there are few absolute contraindications for the procedure including mainly pregnancy, active genitourinary infection, malignancy, and immunosuppression. Relative contraindications are subjective and based on the judgement and experience of the clinician. Large and submucosal fibroids do not appear to be a contraindication to this procedure.

We present a case that shows the consequences of a mismanaged case that unnecessarily increased significantly patient's morbidity and mortality [6, 8–12].

## 2. Case Presentation

**Figure 1:**
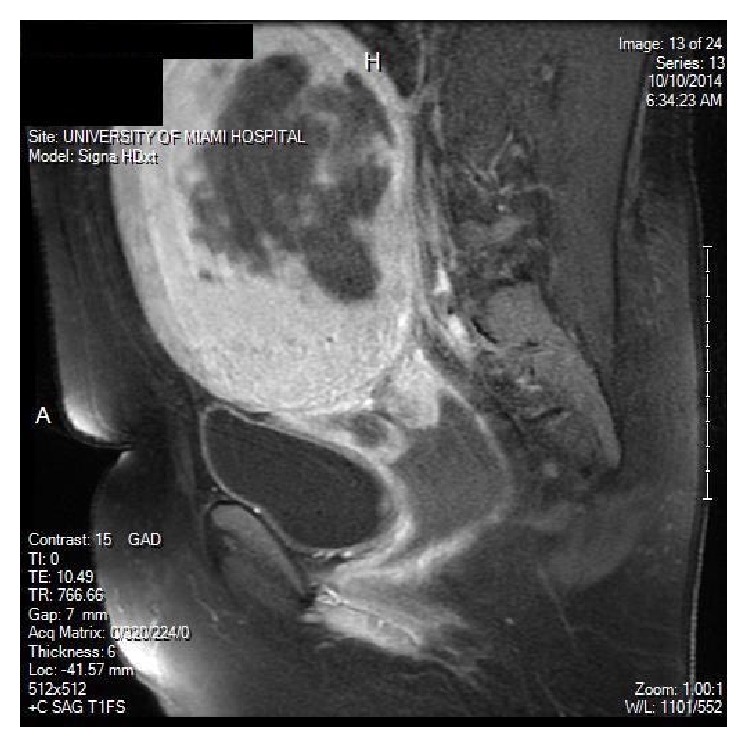
MRI showing a large central necrotic fibroid.

A 44-year-old female with a history of abnormal uterine bleeding and fibroid uterus had a transvaginal ultrasound revealing a 13 × 12 cm intracavitary myoma. Patient had an episode of heavy uterine bleeding, for which she was admitted at an outside facility. There an MRI was performed and confirmed the diagnosis (Figure 1). Uterine artery embolization (UAE) was performed to control the acute bleeding.

**Figure 2:**
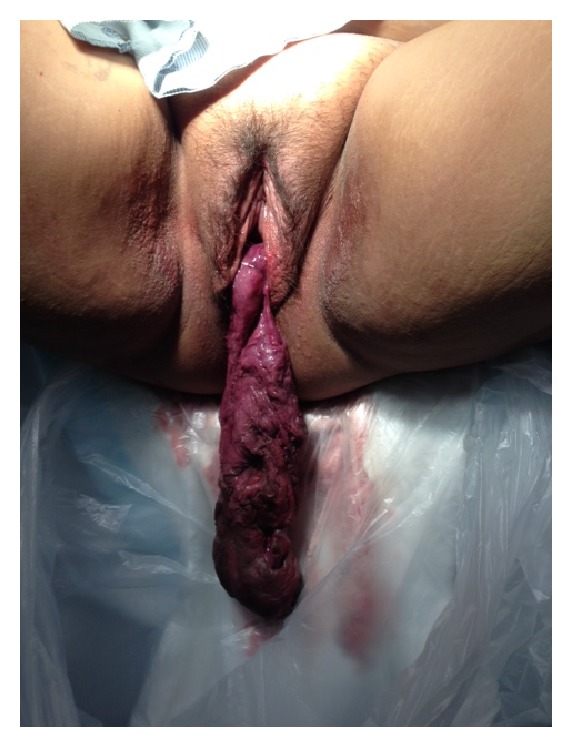
Foul smelling mass protruding from vagina.

Four days after UAE patient presented to our service complaining of pelvic pain, foul smelling vaginal discharge, fever, and a mass protruding from the vagina. On examination a malodorous 15 cm necrotic mass was seen outside the vagina (Figure 2). She was hospitalized and started on IV antibiotics and taken to the operating room and vaginal myomectomy was performed. Uterine cavity was evacuated manually with ring forceps followed by suction curettage. Postoperatively she remained afebrile, was given IV antibiotics for 5 days, and was discharged home on oral antibiotics.

**Figure 3:**
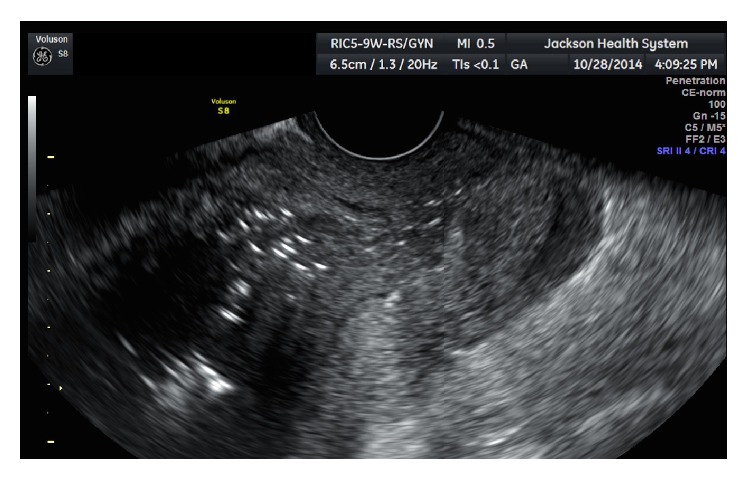
Ultrasound showing gas inside the endometrial cavity.

**Figure 4:**
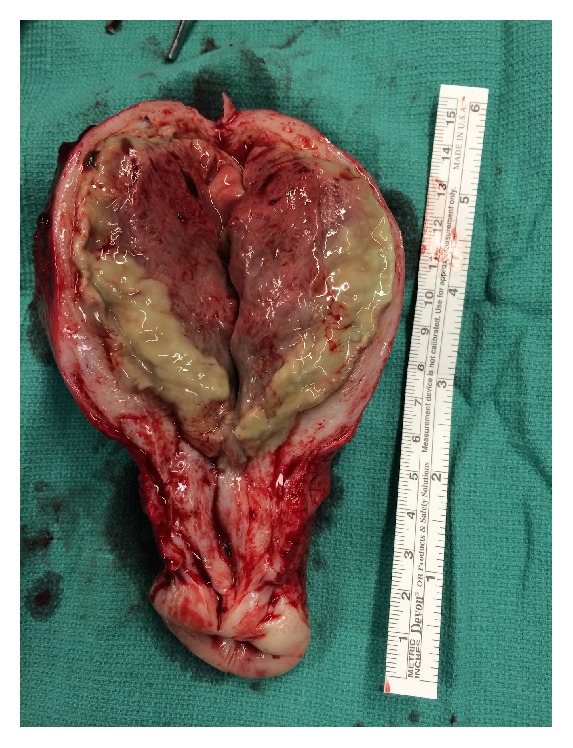
Surgical specimen demonstrating endomyometritis.

One week later she presented to the emergency room with purulent vaginal discharge, bleeding, fever, and elevated white count. Ultrasound revealed gas in the uterine cavity and myometrium (Figure 3). Endomyometritis was suspected and the patient was started on IV antibiotics. Patient continued having fever, and abdominal hysterectomy was performed. Surgical findings and pathology confirmed the diagnosis of uterine necrosis and endomyometritis (Figure 4). Her postoperative course was uneventful.

## 3. Discussion

Uterine artery embolization is an effective alternative treatment to surgical therapy for leiomyomas [16]; however it has limitations. Serious complications are rare after embolization but have been reported in cases of submucosal myomas and especially with fibroids with large dimensions [10, 13, 17]. There are case reports in the literature of sepsis after UAE [9, 10].

Our case reports a solitary large submucosal myoma measuring 13 cm. Early reports have suggested an increased rate of complications when UAE was used to treat fibroids larger than 10 cm [18–21]. However, Bérczi et al. [1] have recently shown that large fibroids do not appear to be a contraindication to UAE.

After reviewing the literature, it seems that the location of the fibroids is the relevant factor related to complications rather than size. Expulsion of the fibroid usually occurs with submucosal and intracavitary fibroids. Verma et al. reported that fibroids with an interface-dimension ratio of 0.55–0.83 and maximum dimension of 3–17 cm on MRI are more likely to become intracavitary and consequently vaginally expelled [16]. In our case where patient had an intracavitary fibroid, the unfavorable interface-dimension ratio could have been used to predict the poor outcome.

Preprocedure MRI has been useful predicting the outcomes of UAE. It allows the differentiation of fibroids regarding size and location providing information that can affect clinical decision. According to Cura et al. [3], MRI has changed the initial diagnosis and treatment plan in 20% of cases being evaluated for UAE. In addition, MRI is also useful to predict who will benefit the most from the procedure [3, 5].

## 4. Conclusion

Uterine artery embolization is a relatively safe procedure for fibroid treatment; however there are no guidelines to determine which fibroids are amenable to embolization regarding their size or location. Submucosal and intracavitary location appear to be more frequently associated with expulsion leading to major complications such as sepsis.

Preprocedure MRI should be performed to improve results and response to treatment. Measurement of the largest endometrial interface seems to have a good reproducibility in determining which fibroids can migrate to the endometrial cavity. Therefore selection of candidates based on this finding is important and patients should be counseled regarding this complication.
